# Determinants of the decision to incision interval in case of emergency caesarean section in Yaoundé’ hospitals

**DOI:** 10.4314/ahs.v22i2.59

**Published:** 2022-06

**Authors:** Pierre Marie Tebeu, Christian Nzentem Tchamte, Nelly Kamgaing, Jesse Saint Saba Antaon, Yvette Nkene Mawamba

**Affiliations:** 1 Faculty of Medicine and Biomedical Sciences, University of Yaoundé I, Cameroon; 2 Department of Gynecology Obstetrics, University Teaching Hospital, Yaoundé-Cameroon; 3 League for Initiative and Active Research for Women's Health and Education (LIRASEF); Cameroon

**Keywords:** Determinant, Decision, Emergency, Caesarean Section

## Abstract

**Objective:**

To analyze the determinants of the decision to incision interval in case of emergency caesarean section in Yaoundé' hospitals

**Methods:**

A prospective cross-sectional (affected / non-affected) study was conducted in four hospitals in Yaoundé between January and may 2017 after National Ethical Committee approval. The target population was women who benefited from emergency caesarean section during the study period. Crude Odds Ratio (OR) and adjusted odds ratio (AOR) with 95% Confidence Interval was used to appreciate the association between several characteristics and the risk for long decision-incision delay.

**Results:**

The overall cases of 165 emergency caesarean section were analyzed. The prevalence of emergency caesarean section performed within 30 minutes was 20%. Social factors associated with long delay to perform the emergency Caesarean section (> 30 minutes) were the primary level of education [ AOR: 3.63(2.44–5.41)], unemployment status [AOR: 5.17(2.95–8.95)]; and the absence of a parent at admission [AOR: 2.2(1.23–3.94)]. Medical factors associated with long delay from decision to incision were: use of spinal anesthesia in opposition to general anesthesia [AOR: 3.86(2.59–5.73)]; delay of transfer from emergency and the late provision of the operation supplies [AOR: 4.18(2.90–6.03)].

**Conclusion:**

Few women benefit from the surgical intervention within a maximum of 30 minutes. Support measures for women presenting the indications for emergency caesarean sections in hospitals are essential to improve the decision-incision delay of the caesarean section emergency.

## Introduction

Cesarean section is a surgical delivery procedure by extracting the fetus after incision of the abdomen (laparotomy) and the uterus (hysterotomy)^1^,^2^. This procedure can be indicated for an elective or an emergency condition. The elective caesarean section is indicated before initiation of labor in absence of immediate materna/fetal threat. The emergency caesarean is performed in case of urgent vital risk for the mother and / or the child. Babies born by emergency cesarean delivery have a higher risk of admission to the neonatology unit, neonatal asphyxia, neonatal infection, preterm birth, and perinatal death^3^. Due to the immediate vital risk for mother and / or the fetus, the time length from decision for cesarean section and delivery is of great importance 2.

The decision-incision delay (DID) is defined as the time interval which extends between the moment when a decision is made to perform the cesarean section and the time of the incision on the abdominal wall^4^,^5^. There is a great heterogeneity on the DID reported in different countries. In Tanzania, a study found that, among 598 women who underwent emergency caesarean section the median Decision Delivery Interval was 60 min and only 12% were operated within 30 min from decision time^6^. A Nigeria one reported that, in two maternities, none of the caesarean sections was done within the recommended 30-min interval^7^. According to another Nigerian study, Among 235 emergency CDs included, only 2.1% occurred within 30 minutes and 86 (36.6%) within 75 minutes. The mean DDI was 119.2±95.0 minutes. A Cameroonian study, found a mean duration to perform emergency caesareans was 224.36 ± 173.30 minutes (≈3 hours and 45 minutes) with extremes of 15 and 864 minutes 8.

Many explicative factors to long DID were reported by authors. The delay recommended by professional organizations varies from 20 to 30 minutes. The delay of 30 minutes had been adopted in Britain and in United States of America 9,10. More Short delay of 20 minutes was adopted in Germany^11^. A Polan study identified factors affecting DDI, including, Obstetrician, Midwives, Anesthesiologists and hospital managers^12^. More specifically, they insisted on the sufficient number of qualified staff, regular training in emergency procedures, availability of operating theatres, as well as fast and safe transportation of patients between the admission room, obstetrics department and operating theatres^12^.

Intervention of those risk factors have shown promised results in reducing the DID^11^,^13^ By reducing the transfer time to the operating room, some authors reported the mean DDI of extremely urgent cesarean significantly decreased from 21.3 minutes 14.9 minutes with increase in the proportion of extreme emergency cesarean sections done in less than 30minutes (85.1% versus 93.5%^14^. In addition, another study carried out in Chad found that 54.9% of emergency Cesarean sections were performed within 30 minutes; this because of the free cesarean section in this country^13^.

Several complications are associated with long DID, hence, in 2013, an Indian study found that when the decision time to give birth exceeds 75 minutes, the neonatal and maternal complications were considerably increased^15^. Another study from Omam, reported the lack of differences in perinatal outcomes for cases with a DDI of ≤30 minutes versus 31–60 minutes were observed; however, a DDI of >60 minutes was significantly associated with poor neonatal outcomes in terms of increased SCBU admissions and low Apgar scores (P <0.001 each)^1^.

The immediate neonatal complications were respiratory distress (37.2%) and neonatal infections (25.6%) increased significantly when the management time was greater than 120 minutes 8. A Cameroonian study identified determinants of prolonged decision-incision period beyond 30 minutes in case of emergency cesarean, including incomplete kit, preoperative assessment unavailable, financial problem, and unavailability of the surgical team. However, this study and no other report these determinants by comparing a control group (with DDI of 30 minutes or less) and a study group (DDI more than 30 minutes) 8. Better knowledge about the determinants of long DID in case of emergency cesarean could help in improving the quality of care and thereby improve the well-being of the mother and the newborn.

## Objective

To analyze the determinants of the decision to incision interval in case of emergency caesarean section in Yaoundé' hospitals, Cameroon.

## Methods

### Study design, site and period

This was a prospective cross-sectional study (affected / unaffected) conducted in four hospitals in Yaoundé, Cameroon between January 05, 2017 and May 31, 2017 after an National Ethical Committee approval(No CEIUDO/1107/07/2017/T). The study sites were, YTH (Yaoundé Teaching Hospital), YCH (Yaoundé Central Hospital), YGOPH (Yaoundé Gynaeco-Obstetric and Pediatric Hospital) and CASS (Centre for Social Animation and Sanitary of Nkoldongo).

### Study population

The target population consisted of women who underwent an emergency caesarean section during the study period, after clear consent. To complete the sample size for our study, we used Kelsey's formula 17. With the proportion of cesarean section performed in an interval of 30 min and more than 30 min 36% and 64% respectively, with 95% confidence interval18. Using STATCALC of the Epi info software version 7.2.2.6, we found the minimum sample size of 153 women (31 cases for affected vs. 122 for non-affected). To increase the power of our results, we recruited the maximum number of subjects who met our inclusion criteria. A total of 165 women with 33 subjects (emergency Cesarean section performed within 30 minutes) and 132 controls. (Emergency Caesarean section performed in more than 30 minutes).

### Variables

Data were collected on socio-demographic, reproductive and clinical characteristics.

Data analysis consisted in comparing the main socio-demographic, reproductive and clinical characteristics among the two study groups, using the Chi-square test for homogeneity or the Fisher exact test for qualitative variables when appropriate.

### Data analysis

All statistical analyzes were performed using SPSS version 23 software. The comparison of quantitative variables was carried out using the Student t test and Chi2 test. To identify factors associated with late caesarean section (after 30 minutes), we used an unconditional logistic regression model. For the multivariate analysis, a step-by-step approach was applied in order to determine the model that were more predicting best predicts the factors associated with performing the late caesarean procedure. Confounding variables used in the multivariate model were selected based on the heterogeneity observed at univariate analysis or with p<0.24 . The level of significance was set up at p<0.05.

## Results

We identified 165 cases of emergency caesarean section in all 4 hospitals, including 33 cases performed within 30 minutes (20%). Thus, 132 Caesarean sections were performed after 30 minutes (80%).

Social factors associated with the long decision-incision delay in performing the emergency caesarean section (more than 30 minutes) were primary level of education [70.13% vs. 94.12%; AOR: 3.63(2.44–5.41), p< 0.001], unemployment [56.52% vs 96.94%; AOR: 5.17(2.95–8.95); p<0.001], and the absence of a parent at admission [73.68% vs 88.57%; AOR: 2.2(1.23–3.94) ; p<0.001]. Medical factors associated with the long decision-incision delay were, the use of spinal anesthesia as opposed to general anesthesia [66.67% vs. 86.16%; AOR: 3.86(2.59–5.73); p <0.001] (Table 1)

**Table 1:** Association between type of anesthesia, socio-demographic characteristics and incision delay

Risk factors	Cesarean realization delay				Crude		Adjusted	
	> 30 min N=132		≤ 30 min N=33		OR (CI 95%)	P-Value	AOR(CI95%)	P-value
	n	%	n	%				
**Age (year old)**								
**Age (Median)**	26 (16 – 42)	27 (22 – 36)						
**Age (Class)**								
[15–20[	19	100	0	0.00	-			
[20–30[	77	81.05	18	18.95	1.89 (0.85–4.18)	-	-	-
[30–46[	36	70.59	15	29.41	1^c^			
**Study level**								
Primary	32	94.12	2	5.88	6.81 (1.50–30.84)	0.001	3.63(2.44–5.41)	<0.001
Secondary	46	85.19	10	18.51	1.95 (0.85–4.69)	0.1133		
Higher level	54	70.13	23	29.87	1^c^			
**Marital status**								
Married	64	74.42	22	25.58	1^c^			
Single	68	86.08	11	13.92	2.13 (0.95–4.43)	0.0614	-	-
**Profession**								
Have a work	39	56.52	30	43.48	1^c^			
Unemployed	95	96.94	3	3.06	24.35 (7.02–84.51)	0.0001	5.17(2.95–8.95)	<0.001
**Absence of parents**								
Yes	62	88.57	8	11.43	2.77 (1.16–6.58)	0.02	2.2(1.23–3.94)	<0.001
No	70	73.68	25	26.32	1^c^			
**Type of anesthesia**								
General Antestthesia	34	66.67	17	33.33	1^c^			
Spinal Anesthesia	85	86.16	16	14.84	2.86 (1.21–5.85)	0.013	3.86(2.59–5.73)	<0.001
Conversion	13	100	0	0.00				

The delay in transportation from the obstetric emergency room to the operating room of more than two minutes [25.81% vs 94.12%; AOR: 7.72(5.16–11.56); p<0.001] and the delay in providing the operating kit (supplies) (10–30 minutes)[32.61% vs 93.55%; AOR: 4.18(2.90–6.03); p <0.001] (Table 2).

**Table 2:** Association between the times for performing the caesarean section and the factors likely to delay the time for treatment

Risks factors	Caesarean realization delay				*crude*		*Adjusted*	
	> 30 min N=132		≤ 30 min N=33		*OR (CI 95%)*	P-Value	AOR(*(CI 95%)*	P value
	n	%	n	%				
Transfer delay to theatre								
<2min	8	25.81	23	74.19	1^c^			
2–5min	112	94.12	7	5.88	46.00 (15.17–139.47)	<0.0001	7.72(5.16–11.56)	<0.001
>5min	12	80	3	20	4.0 (0.91–17.53)	0.499		
**Complete kit**								
Yes	85	77.98	24	22.02	1^c^			
No	47	83.93	9	16.07	1.48 (0.63–3.43)	0.3658	-	-
**Delivery delay of kit**								
<5min	3	100	0	0.00	-			
5–10min	15	32.61	31	67.39	1^c^			
10–30min	29	93.55	2	6.45	29.97 (6.29–142.50)	<0.0001	4.18(2.90–6.03)	<0.001
NA*	85	100	0	0.00				

* NA : Not Applicable

## Discussion

We conducted a cross-sectional study on the determinants of the long decision-incision delay for an emergency caesarean in Cameroon. We found that 20% of emergency caesarean section was performed within 30 minutes. In the literature, the frequency of emergency caesarean sections performed within this period varied between 0% and 54.9% 15, 13,19- 24,6. Some authors have reported a frequency higher than ours, thus, we find 49% and 54.9% in French and Chadian studies respectively 19,20. Others found low frequencies ranging from 0% and 8%. In a Nigerian study from which, no emergency caesarean section was performed within 30 minutes 7. Some authors had reported the proportion of emergency caesareans almost similar to ours, 19% 21.

The small proportion of early performed caesarean section (20%) resulting from our research, although higher than those reported by certain research teams highlight the need to implement strategies likely to allow the realization of caesarean section within 30 minutes. These strategies may include the availability of KITS (supplies) in delivery rooms, and raising awareness of women of childbearing age about high birth.

Regarding the level of education, those who did not reach the secondary level were most often operated late (24.24% vs 6.06%). Thus, the fact of not having reached the secondary level of education was associated with an increase of the risk of long decision-incision delay [AOR = 3.63 (2.44–5.41)]. In the literature, we did not find a report on this association. However, other studies in reproductive health had already shown the effect of the low level of education on maternal health. Hence, risk of poor knowledge on obstetric fistulas was shown as doubled in absence of formal education 25. The low level of education associated with late caesarean delivery may be explained by the fact that women with a low level of education may have inappropriate information about birth preparedness. Moreover, low level of education may limit the level of good knowledge concerning to caesarean section and related complications. Accordingly, the delay in obtaining consent could extend the interval for caesarean delivery. Ours findings highlight the need to implement communication strategies on safe delivery targeting women with low education level.

Regarding the profession, we found that 59.39% of the study participants did not have an occupation.

We found that, unemployed women were more likely to be operated after 30 minutes. The lack of employment as a factor associated with the non respect of the interval recommended by the World Health Organization can be explained by the fact; these women would not have financial means for the acquisition of KIT. Consequently, they would have delayed their arrival at the hospital, waiting for the help from a partner or another relative to ensure the intervention related fee. Some research work carried out in the city of Yaoundé-Cameroon (i.e.: in particular at the gyneco-obstetric and pediatric hospital of Yaoundé), had reported a similar result with the patient income as a determinant for delay for early performing emergency cesareans) 25. Similarity of findings from those two studies could be explained by the similarity of the study settings. These results emphasize on the need for improving the accessibility to caesarean by free of charge service or cost sharing. We found that in comparison to general anesthesia spinal anesthesia was frequently used in late operated patients. Our findings could be explained by the fact that spinal anesthesia requires more preparation time than general anesthesia Swedish; Benin and Indian studies had similar results^26^, ^20^, ^23^.

The absence of a parent at the time of the decision was associated with a higher risk of late caesarean section. This can be explained by the fact that consent is often conditioned by the parent's opinion. A study in the Republic of Chad, showed that a delay of more than 30 minutes was linked to the absence or refractory attitude of the family about caesarean section for 80 % of cases 13.Concerning the transfer from delivery room to the operation room, once the operation decision taken, the delay of more than two minutes is associated to long incision delay. A French study also identified the delay in transfer to the operating room as one of the causes of late emergency caesareans 19. A Swedish study showed that the 30-minutes caesarean section was due in part to the fact that the access time to the operating room from the obstetric emergency room was 30 to 60 seconds 7. In our case and according to our observations during the research, we found that when the team was well organized (availability of the surgeon, parents and operating kit, etc.), the patient was immediately taken to the operating room and surgery was immediately performed; but, when the surgeon was not available quickly or when the parent was still to come, the delay of transfer was long.

The late provision of the operating kit (10–30 minutes) was associated with a long decision-incision delay in performing caesarean section. This observation can be explained by the fact that the operating supplies are essential for performing a caesarean section and this was not always available, and when available, it was not complete. In the absence of a complete KIT, the patient parent had to go around the city or in pharmacies to look for supplies to constitute the kit prior to surgery. Our results is supported by that from Chad, which revealed that delay of more than an hour in the time for performing the caesarean is partly due to the incomplete Kit despite the free caesarean in their context 13.

## Conclusion

The rate of emergency caesarean delivery within a reasonable time is 20%. The social factors associated factors to the delay in performing the caesarean section are: low level of education unemployment and absence of a parent at admission. Medical factors were, late patient's transfer from the maternity to the operating room (> 2 minutes) and the use of spinal anesthesia. We must act on these factors if quality of the caesarean section is to be improved on.
